# Npvf: Hypothalamic Biomarker of Ambient Temperature Independent of Nutritional Status

**DOI:** 10.1371/journal.pgen.1005287

**Published:** 2015-06-12

**Authors:** Julia Jaroslawska, Agnieszka Chabowska-Kita, Monika M. Kaczmarek, Leslie P. Kozak

**Affiliations:** Institute of Animal Reproduction and Food Research, Polish Academy of Sciences, Olsztyn, Poland; Technische Universität München, GERMANY

## Abstract

The mechanism by which mice, exposed to the cold, mobilize endogenous or exogenous fuel sources for heat production is unknown. To address this issue we carried out experiments using 3 models of obesity in mice: C57BL/6J+/+ (wild-type B6) mice with variable susceptibility to obesity in response to being fed a high-fat diet (HFD), B6. *Ucp1-/-* mice with variable diet-induced obesity (DIO) and a deficiency in brown fat thermogenesis and B6. *Lep-/-* with defects in thermogenesis, fat mobilization and hyperphagia. Mice were exposed to the cold and monitored for changes in food intake and body composition to determine their energy balance phenotype. Upon cold exposure wild-type B6 and *Ucp1-/-* mice with diet-induced obesity burned endogenous fat in direct proportion to their fat reserves and changes in food intake were inversely related to fat mass, whereas leptin-deficient and lean wild-type B6 mice fed a chow diet depended on increased food intake to fuel thermogenesis. Analysis of gene expression in the hypothalamus to uncover a central regulatory mechanism revealed suppression of the *Npvf* gene in a manner that depends on the reduced ambient temperature and degree of exposure to the cold, but not on adiposity, leptin levels, food intake or functional brown fat.

## Author Summary

Current knowledge does not provide a clear, definite view of central mechanisms controlling energy balance upon cold-activated thermogenesis. Here we show that upon cold exposure lean mice maintain body composition but increase food intake to fuel thermogenesis, whereas cold-exposed mice with DIO utilize endogenous fat stores and then transition to increased food intake as body composition approaches that of the lean controls. Using knockout mice with leptin and *Ucp1* gene deficiency our study indicates that the relative energy utilization from food intake and endogenous energy reserves to maintain body temperature during cold exposure is independent of both leptin action and brown fat-linked thermogenesis. Using a combination of genetic and biological approaches, we demonstrate that *Npvf* gene expression in the hypothalamus is regulated by changes in ambient temperature in a manner independent of the nutritional status of the mouse.

## Introduction

Reduced ambient temperature will increase thermogenesis and reduce obesity. However its long-term effectiveness as a strategy to reduce obesity has been questioned because of the expectation that increased energy expenditure for the cold environment will increase food intake, thereby neutralizing the weight reducing effects of the cool environment [1], a skepticism also associated with the effectiveness of physical activity as an anti-obesity strategy [2]. This skepticism emerges from the adipostat hypothesis itself, which predicts that reductions in fat mass by cold stimulation will be compensated by increased food intake to maintain its adiposity index [3]. On the other hand, studies on loss of fat mass by increasing thermogenesis with the chemical uncoupler dinitrophenol (DNP) showed that increased food intake does not necessarily occur [4]. Therefore compensation as predicted by the adipostat model may also not occur in association with BAT thermogenesis. Since chemical uncoupling by DNP, or even activation of thermogenesis by adrenergic receptor agonists [5], are unregulated inductions of thermogenesis, compared to normal physiological mechanisms regulating body temperature, the problem of predicting the effectiveness of achieving energy homeostasis from food intake and endogenous energy reserves during cold exposure remains. Specifically, when an individual is exposed to a cold environment how the physiological decision is made to use endogenous energy reserves or to increase food intake and how this decision is influenced by the obese state of the individual is unknown.

Although significant recent research progress has enhanced our understanding of the central control of BAT thermogenesis and energy expenditure in cold-exposed mammals, some areas are yet not well understood. In cold-exposed animals increased thermogenesis is associated with increased feeding, but is not accompanied by a gain of weight [6]. Coordinated increases in thermogenesis and food intake during cold exposure are controlled by signaling events in hypothalamus that are undefined. Within the hypothalamus, only a few genes are known to be differentially regulated in response to reduced ambient temperature [7–12], but one cannot identify a clear pattern of neuropeptide expression characteristic for the hypothalamic response to the cold. The contribution of the selective neuro-hormone systems such as NPY or TRH in the regulation of cold-activated thermogenesis and feeding behavior has been extensively studied using pharmacologic approaches [13,14] or animal knockout models [15–17]. However, neither of these approaches identifies a critical molecule or describes signaling events that account for central mechanisms controlling energy availability and utilization under cold conditions.

In this study, using wild-type B6 and brown fat deficient *Ucp1-/-* mice with DIO and genetically obese (*Lep-/-*) mice, we first determined that cold-induced thermogenesis is preferentially fueled by oxidation of fat reserves in individuals with environmental obesity and by food intake in lean individuals. We then analyzed global gene expression in the hypothalamus of cold-exposed mice and found that suppression of *Npvf* neuropeptide precursor mRNA levels occurred in the three models of obesity. To our knowledge *Npvf* is the only transcriptional target in hypothalamus known to be selectively regulated by changes in ambient temperature.

## Results

### Experiment 1: Energy expenditure during cold exposure of mice with DIO

**Fig 1:**
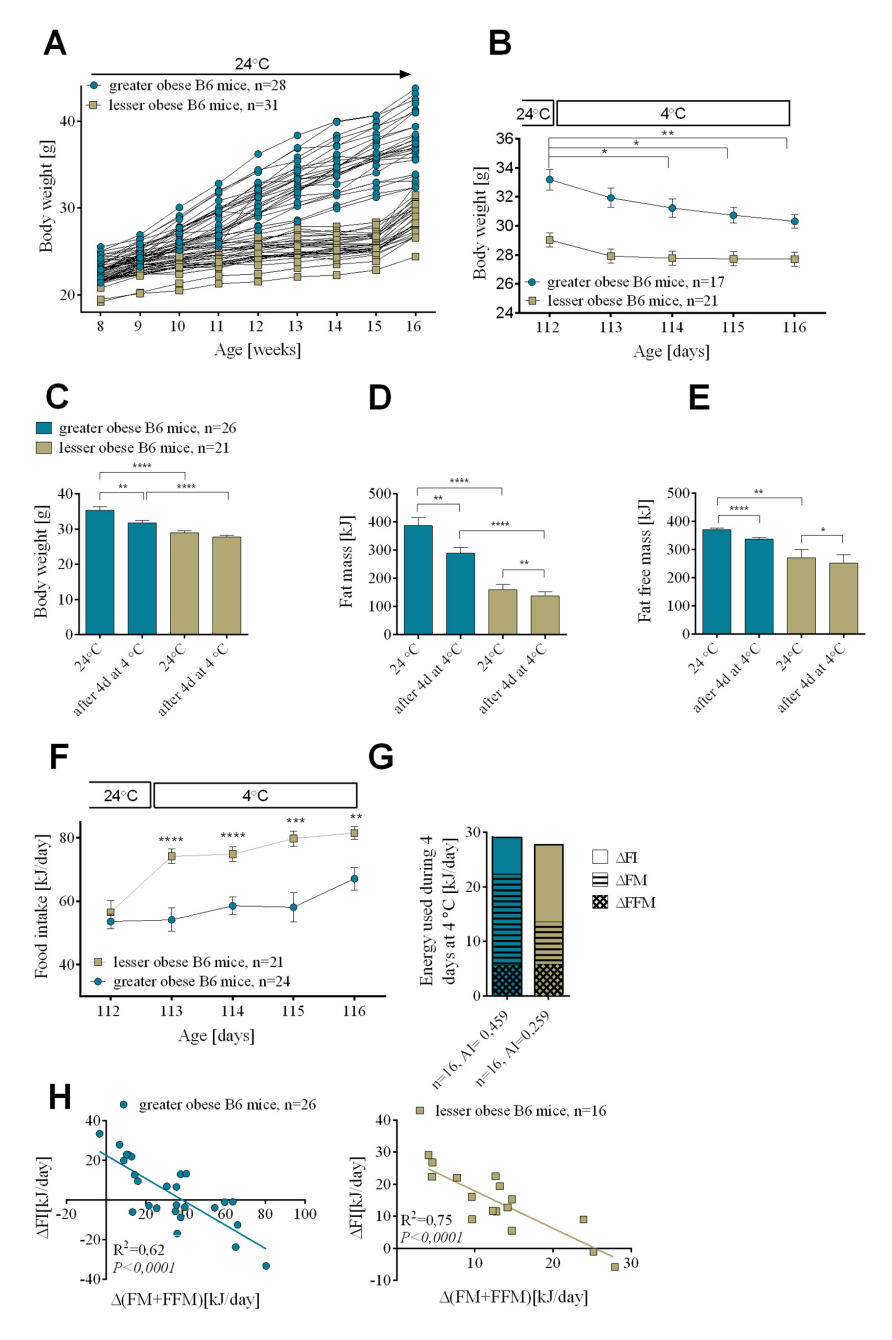
Changes in endogenous substrate utilization and food intake associated with cold-induced thermogenesis in mice with variable levels of DIO. Diet-induced increase in body weight (A). Reduction in body weight during cold exposure (B). Changes in body weight (C), fat mass (D), and fat free mass (E) before and after 4 days at 4°C. Daily changes in food intake during 4 consecutive days at 4°C (F). Comparison of energy utilization from endogenous reserves and food intake in greater obese and lesser obese B6 mice (G). Correlations between daily increase in food consumed and internal body reserves mobilized per day during 4 days in the cold (H). Data are expressed as mean ± SEM. *, significant differences (*t* test, *, *P* < 0.05; **, *P* < 0.01; ***, *P* < 0.005; ****, *P* < 0.001). FI, food intake; FM, fat mass; FFM, fat free mass.

A wide range of body weight in genetically identical B6 mice results from their high natural variation in susceptibility to DIO [18]. We utilized this variation together with feeding mice a HFD for different lengths of time to generate mice with a range of adiposity. After 8 weeks or 1 week of feeding a HFD a cohort of mice was produced in which body weight ranged between 32.4 and 43.8g (greater obese mice) and between 24.4 and 32.7g (lesser obese mice) (Fig 1A). Reducing the ambient temperature from 24 to 4°C resulted in an immediate lowering in body weight that was highest on day one and gradually diminished during the succeeding days (Figs 1B and S1A). Although lesser and greater (range of body weight) obese mice showed the same response, the weight loss was larger in the greater obese group than the lesser obese group (Figs 1B, 1C, S1A and S1B). Fat mass was the major endogenous substrate fueling thermogenesis (Figs 1D and S1B). In the greater obese group, after 4 days at 4°C 97.5 kJ of energy came from fat mass and 33.8 kJ from fat free mass. For the lesser obese group, 30 kJ came from fat mass and 22.9 kJ from fat-free mass. Thus, 4 days of cold exposure resulted in total use of endogenous energy that equaled 131.3 kJ for the greater obese mice and only 52.9 kJ for the lesser obese mice (Fig 1D and 1E). After one day at 4°C both groups of mice experienced a slight decline in body temperature (1–2°C), however, by the 2^nd^ day at 4°C all mice were able to thermoregulate and maintain their body temperature at the level at which they started (36 ± 1°C).

If the lesser obese group utilized less of their endogenous energy reserves during cold exposure than the greater obese, then where did the energy for thermogenesis come from? For this we measured food intake. After 16 weeks on the dietary regime at 24°C, as described in the Methods, food intake was 56.5 ± 3.64 kJ/day for the lesser obese and 53.6 ± 2.29 kJ/day for the greater obese (Fig 1F). When mice were transferred to 4°C, food intake immediately increased in the lesser obese mice to 75 kJ/day (35% increase) and to 84 kJ/day (50% increase) after 1 and 4 days, respectively; the increase in food intake was smaller in the greater obese mice going to 55 kJ/day (2% increase) and 67 kJ/day (25% increase), respectively, after 1 and 4 days in the cold. With increasing time at 4°C the difference in food intake between the lesser and greater obese groups was reduced (S1C Fig), consistent with the diminishing difference in fat mass. After the first day of cold exposure the difference in food consumption between mice from 2 cohorts equaled 20.06 ± 4.53 kJ, after 4 days at 4°C it was 14.44 ± 4.23 kJ (Fig 1F) and only 9.53 ± 2.87 kJ after 7 days at 4°C (S1C Fig). Cold-induced thermogenesis is associated with increased consumption of fuel reserves and, as evident in Figs 1F and S1C, mice with lower endogenous fuel reserves compensate by increasing food intake, a process that apparently increases with time as endogenous fuel reserves become depleted. After 4 days at 4°C, regardless of the level of obesity present in the animals before cold exposure, cumulative energy coming from feeding and mobilized endogenous energy stores was comparable in the greater and lesser obese mice (Fig 1G).

For both lesser and greater obese animals linear regression analysis revealed a strong negative correlation between energy reserves (fat and fat free mass) mobilized per day and daily energy consumed during time spent in the cold (R^2^ = 0.62 for greater obese mice and R^2^ = 0.75 for lesser obese after 4 days in the cold) (Fig 1H). An equally strong negative relationship was observed when values of adiposity index calculated for each mouse before cold exposure were plotted against daily food intake during 4 days at 4°C (R^2^ = 0.74 for both greater and lesser obese mice) (S1D Fig).

An important observation is that mice with robust DIO after 8 weeks on a high fat diet at 24°C will concurrently increase food intake and reduce body weight when transferred to an ambient temperature of 6°C (S2A and S2B Fig). They will stabilize both body weight and food intake to a new state of energy balance to maintain body temperature. When they are returned to 24°C food intake returns to the level observed before the cold exposure and they resume the increase in adiposity characteristic of B6 mice.

### Leptin status and the cold challenge

**Fig 2:**
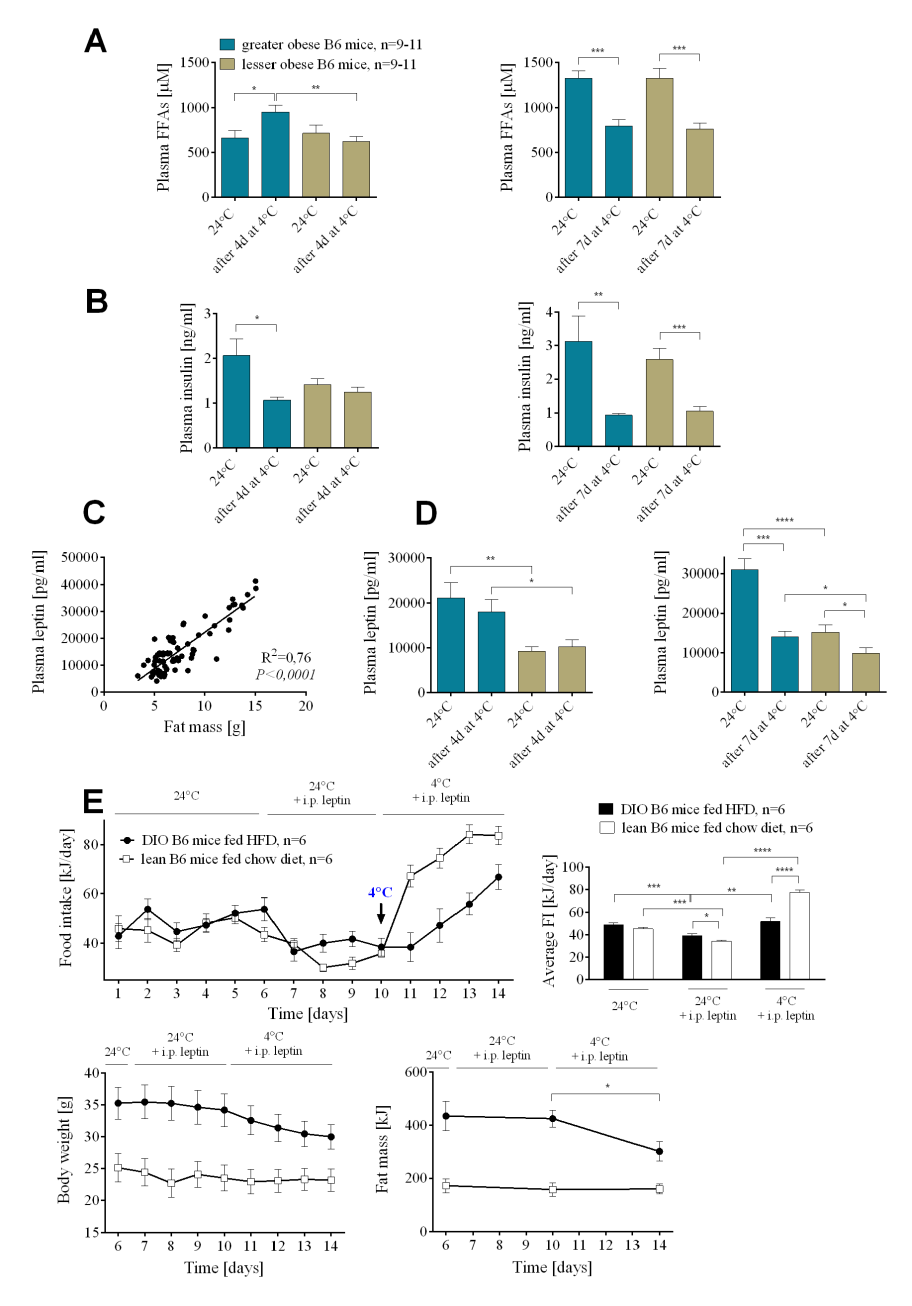
Insulin and leptin resistance in wild-type B6 DIO mice. Changes in plasma free fatty acids (A) and insulin (B) before and after 4 and 7 days at 4°C in greater and lesser obese B6 mice. Correlation between fat mass and plasma leptin levels in DIO mice (C). Changes in plasma leptin (D) before and after 4 and 7 days at 4°C in DIO mice. Changes in food intake and body weight and composition (E) in mice administered with leptin at 24 and 4°C. Data are expressed as mean ± SEM. *, significant differences (*t* test, *, *P* < 0.05; **, *P* < 0.01; ***, *P* < 0.005).

At 24°C there were no differences in the level of plasma free fatty acids (FFAs) and insulin between the groups (Fig 2A and 2B). After 4 days at 4°C, greater obese mice had significantly elevated levels of circulating FFAs in comparison to lesser obese, consistent with increased fat mobilization in the greater obese animals. Substantial fat mass loss after 7 days of cold exposure resulted in reduced plasma FFAs in both groups of mice. Similarly, after 7 days at 4°C, circulating insulin was decreased in greater and lesser obese mice compared to 24°C (Fig 2B). At 24°C leptin levels were positively correlated with adiposity (Fig 2C). Leptin levels did not drop during the first 4 days at 4°C, only after 7 days in the cold did highly significant reductions in leptin levels occur (Fig 2D).

We evaluated the effects of leptin administration to DIO B6 mice fed HFD or lean B6 mice fed chow diet on the food intake and utilization of endogenous energy substrates before and after cold challenge. Leptin administration at 24°C decreased average daily food intake from 49.12±1.68 to 39.15±1.69 kJ in DIO mice and from 45.40±1.55 to 34.30±1.21 kJ, in chow fed lean mice (Fig 2E). There was no effect of leptin administration on either body weight or body composition of mice at 24°C (Fig 2E). When the ambient temperature was reduced from 24 to 4°C lean mice receiving leptin immediately increased food intake, whereas their body weight and fat mass did not change. On the other hand, cold-exposed and leptin-administered DIO mice immediately utilized endogenous reserves, then as these reserves diminished, they increased food intake (Fig 2E). These results on food intake and fat utilization with leptin administration are not different from the phenotypes in the absence of exogenous leptin (Fig 1B and 1F).

### Experiment 2 Part (a): The effects of leptin deficiency on cold-induced energy expenditure

**Fig 3:**
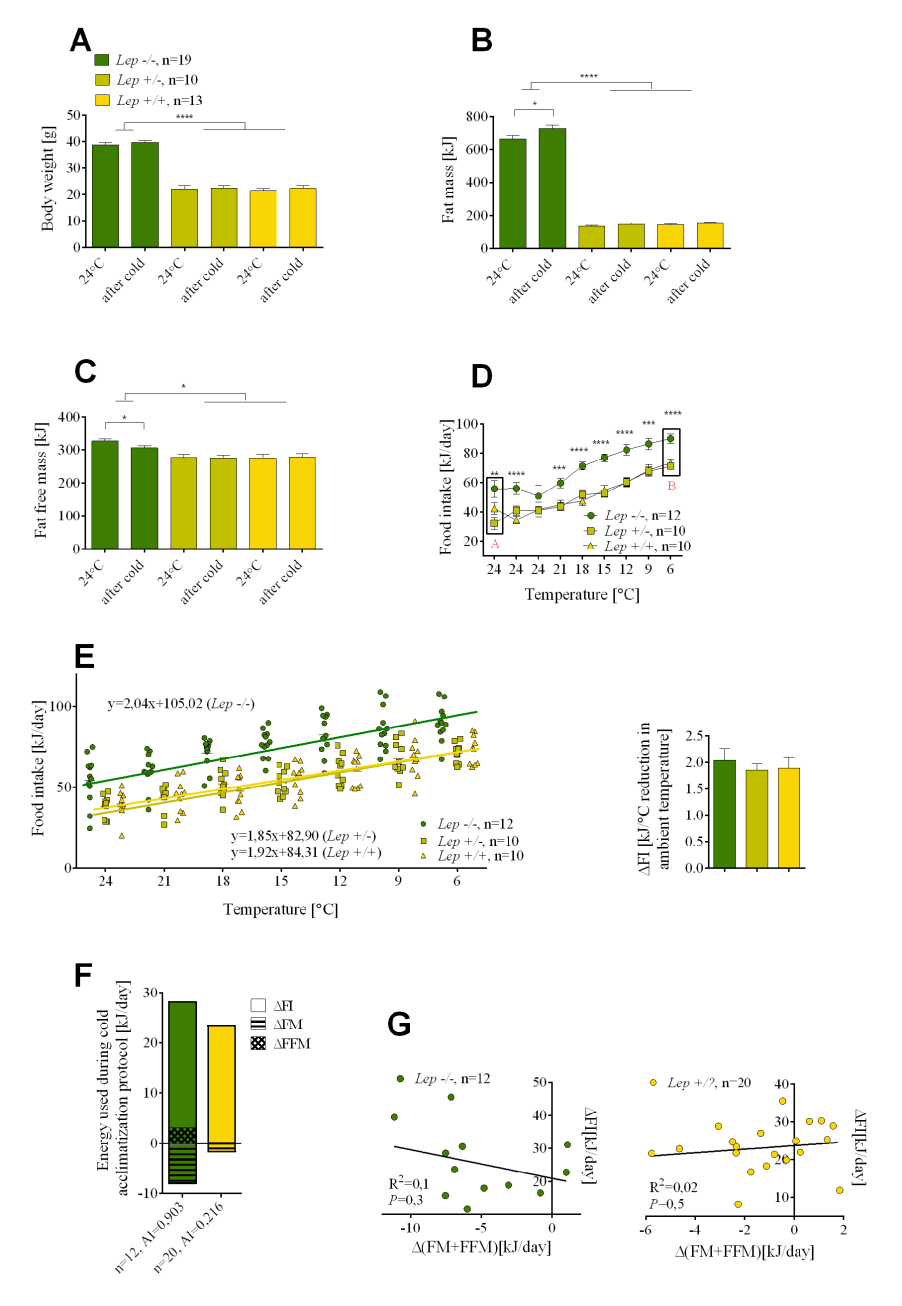
The effects of leptin deficiency on the relative utilization of food intake and endogenous energy stores during cold-induced energy expenditure. Changes in body weight (A), fat mass (B), fat free mass (C), and food intake (D) determined before and after cold adaptation protocol in *Lep-/-*, *Lep+/-* and wild-type *Lep+/+* mice. The rate of an increase in food intake per degree Celsius reduction in ambient temperature in mutant *Lep-/-* and control mice (E). Comparison of energy utilization from endogenous reserves and food intake in mutant *Lep-/-* and *Lep+/*? controls (F). Correlations between daily increase in food consumed and internal body reserves mobilized per day in the cold (G). Data are expressed as mean ± SEM. *, significant differences between mice (*t* test, *, *P* < 0.05; **, *P* < 0.01; ***, *P* < 0.005; ****, *P* < 0.001). FI, food intake; FM, fat mass; FFM, fat free mass.

Mice deficient in either leptin or the leptin receptor are cold intolerant when acutely exposed to 4°C; however, they are able to adapt to a lower temperature if the exposure is gradual [19–21], thereby enabling an analysis of energy utilization during a cold challenge. Although there were large differences in body mass and composition between *Lep+/*? and *Lep-/-* mice fed a low fat chow diet at 24°C, after 9 days in the cold neither genotype showed significant changes in body weight mass nor composition (Fig 3A–3C).

With no reduction in endogenous energy reserves we looked to an increase in food intake. At 24°C average daily food intake was about 40% higher in leptin-deficient than in the *Lep+/*? control mice, as previously observed by Coleman [20] (Fig 3D). One would anticipate that this source of energy would be used to fuel thermogenesis, however, reducing the ambient temperature by 3°C per day resulted in an increase in food intake in both control and *Lep-/-* mice. This food intake curve is displaced upward by an amount corresponding to the difference in food intake between control *Lep+/*? and *Lep-/-* mice at 24°C (Fig 3D). Therefore, the rate of increase in food intake per degree Celsius reduction in ambient temperature by the control mice and *Lep-/-* mice was essentially indistinguishable (Fig 3E). The most striking observation was that *Lep-/-* mice, already hyperphagic at 24°C, further increased their food intake under a cold challenge. After correcting for the slight changes in body composition that occurred in mice upon cold exposure, the total energy used for cold-induced thermogenesis was equal in leptin-deficient and control mice (Fig 3F). Accordingly, there were no significant correlations either for control lean *Lep+/*? or for obese *Lep-/-* mice between the daily increase in food intake and endogenous body fuel reserves mobilized per day in the cold (Fig 3G), in contrast to the significant correlations in DIO mice (Fig 1H).

### Experiment 2 Part (b): The effects of UCP1 deficiency on cold-induced energy expenditure

**Fig 4:**
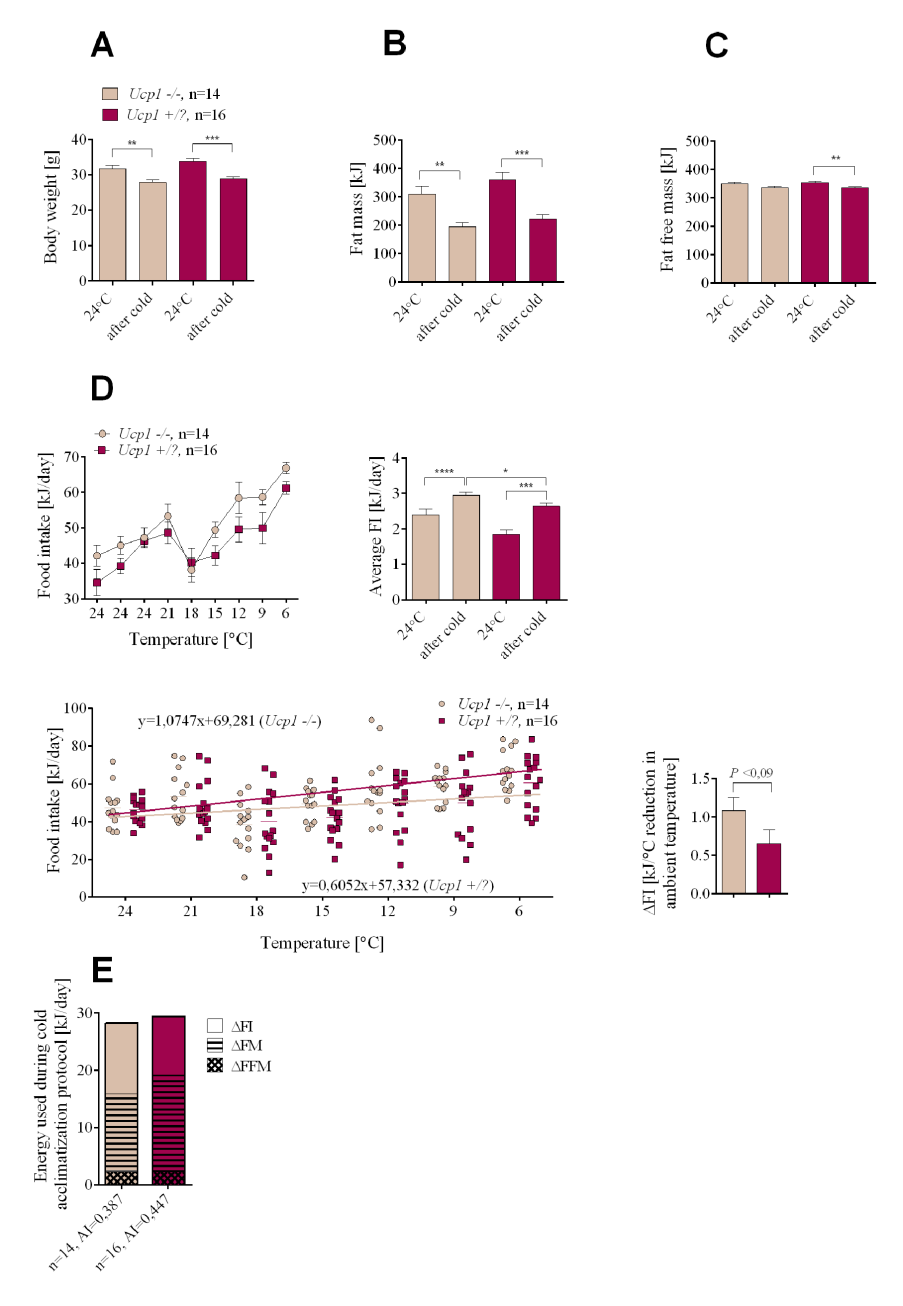
Changes in endogenous substrate utilization and food intake in cold-exposed *Ucp1-/-* and *Ucp1+/*? mice with DIO. Changes in body weight (A), fat mass (B), fat free mass (C), and food intake (D) measured at normal ambient temperature (24°C) and during the cold adaptation protocol. Comparison of energy utilization from endogenous reserves and food intake in *Ucp1-/-* and *Ucp1+/*? controls (E). Data are expressed as mean ± SEM. *, significant differences between mice (*t* test, *, *P* < 0.05; **, *P* < 0.01; ***, *P* < 0.005; ****, *P* < 0.001). FI, food intake; FM, fat mass; FFM, fat free mass.

It is assumed that non-shivering thermogenesis of brown fat is essential for providing the heat to protect the animal from the cold. Indeed *Ucp1-/-* newborn mice on either the B6 and 129 genetic backgrounds cannot survive the first days of birth in a breeding room maintained at ~23°C and *Ucp1-/-* adult mice acutely exposed to the cold at 4°C will succumb within 5 hours [22,23]. However, similar to *Lep-/-* mice, *Ucp1-/-* mice can adapt to the cold [24]. *Ucp1-/-* and *Ucp1+/*? mice were exposed to the cold using the same protocol as that used for *Lep-/-* mice, except that DIO was first induced at 24°C as with the greater and lesser obese mice (Fig 1A). The level of obesity for the *Ucp1+/*? resembled that of the greater obese B6.*+/+* mice, whereas the *Ucp1-/-* mice resembled the lesser obese mice (Fig 4A–4C), even though they were fed the HFD for the full 8 weeks. This is expected, since at 24°C *Ucp1-/-* mice are resistant to DIO [23]. At 24°C food intake was similar for mutant and control mice, whereas the daily energy intake during cold adaptation was higher for *Ucp1-/-* mice (Fig 4D). Similar to the results of the initial experiment with wild type B6 mice, *Ucp1+/*? mice which had the greater obese phenotype preferentially lost fat mass during cold adaptation, whereas the *Ucp1-/-* mice which had the lesser obese phenotype preferentially increased food intake (Fig 4E). Thus, energy balance and substrate utilization in DIO *Ucp1-/-* mice during cold exposure resembles that of lesser obese wild-type mice.

In summary, UCP1-dependent brown fat thermogenesis is not required to derive the weight reducing benefits of adapting to the cold and there is no mechanism associated with thermogenesis that will increase food intake of the greater obese to preserve the obese state. There is a mechanism, however, to preserve a minimal adiposity index typified by young adult C57BL/6J mice fed a low fat chow diet. Total energy consumption as shown by 6 experimental groups (Figs 1G, 3F and 4E) indicates that energy expenditure during cold exposure is generally similar, except that *Ucp1-/-* mice are metabolically inefficient and have higher O_2_ consumption per mouse [25]. The difference among groups describes source of energy for the induction of thermogenesis, endogenous reserves vs food intake, and it is this difference which is the focus of this study.

### A molecular pathway associated with cold activated thermogenesis

**Fig 5:**
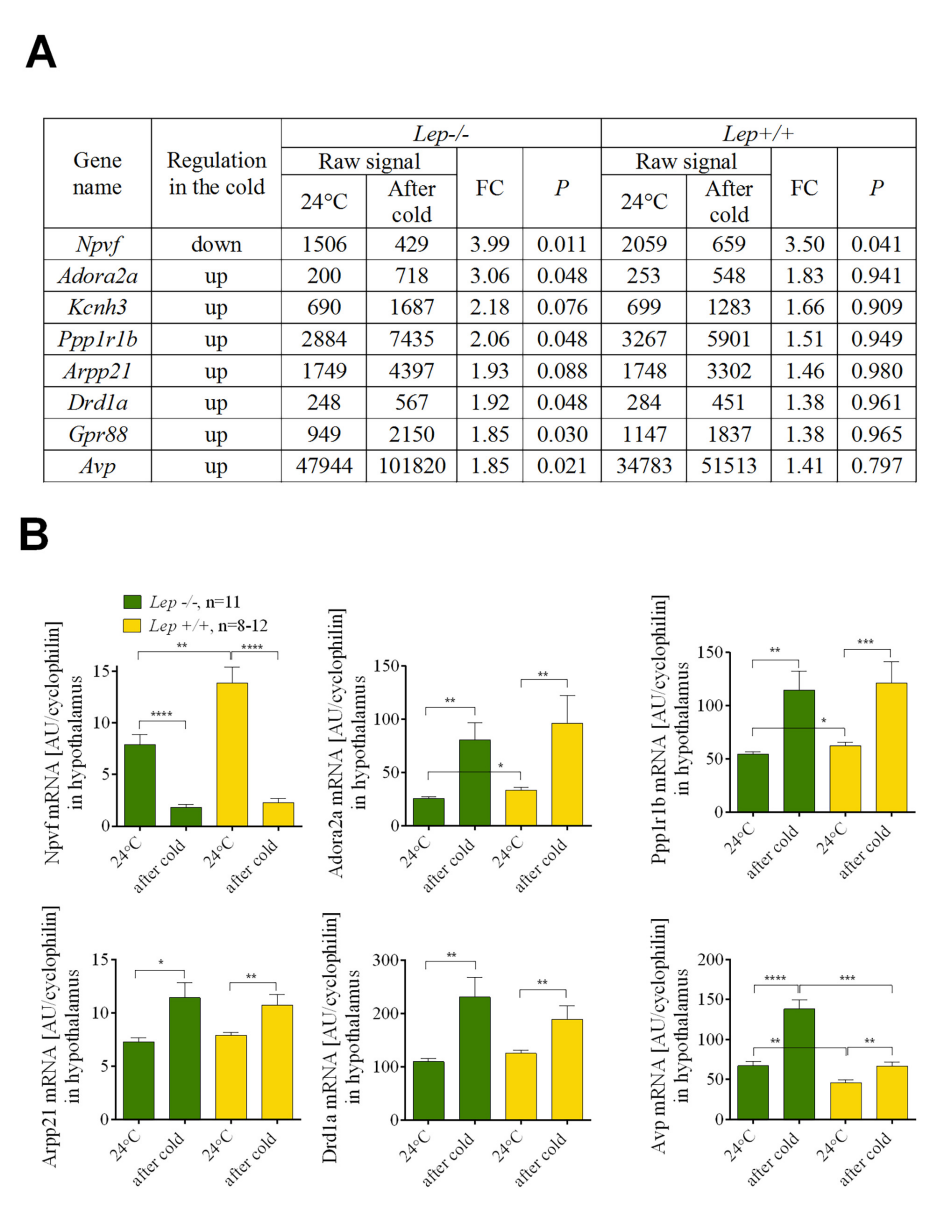
*Npvf* gene is a hypothalamic biomarker of cold-activated thermogenesis. List of the genes that were similarly regulated upon cold exposure in *Lep-/-* and wild-type *Lep+/+* mice (A). Verification of microarray data using qRT-PCR in *Lep-/-* and wild-type *Lep+/+* mice (B). Data are expressed as mean ± SEM. *, significant differences between mice (*t* test, *, *P* < 0.05; **, *P* < 0.01; ***, *P* < 0.005; ****, *P* < 0.001). Fold change (FC) was calculated based on normalized signal values.

At 24°C *Lep-/-* mice are hyperphagic compared to the *Lep+/+* or *Lep+/-* mice (Fig 3D). Reducing the ambient temperature from 24 to 6°C was accompanied by a graded parallel increase in food intake, corresponding to approximately 50 kJ of energy for both control and mutant mice (Fig 3D). Consequently, the same leptin-independent increase in food intake was observed during the transition from 24 to 6°C in both *Lep+/+* and *Lep-/-*. Since the energy content of *Lep+/+* and *Lep-/-* mice was unchanged during cold exposure, thermogenesis is fueled solely by food intake. Accordingly, we predicted that the same changes in gene expression associated with the central regulation of thermogenesis by the hypothalamus must occur in both *Lep+/+* and *Lep-/-* mice during the transition from 24 to 6°C. Microarray analysis of gene expression was performed on hypothalamic tissue dissected from *Lep-/-* and *Lep+/+* mice kept at different temperature conditions, that is, in mice maintained at 24°C (point A, Fig 3D) and in mice in which the ambient temperature had been reduced to 6°C (point B, Fig 3D). We identified a small subset of genes in *Lep-/-* in common with *Lep+/+* mice during the transition from 24 to 6°C (Fig 5A). Among these genes, neuropeptide VF precursor (*Npvf*), showed a robust down-regulated expression of 4.0 and 3.5 fold in the hypothalamus of cold-exposed *Lep-/-* and *Lep+/+*, respectively. A group of genes encoding for G protein-coupled receptors (GPCRs) including the dopamine receptor D1 (*Drd1a*), adenosine receptor 2A (*Adora2a*), GABA(A) receptor subunit delta (*Gabdr*) and *Gpr88* as well as some of their downstream targets including cAMP-regulated phosphprotein 21 (*Arpp21*) and protein phosphatase 1 regulatory subunit 1B (*Ppp1r1b*) were up-regulated 1.4 to 3 fold in both *Lep+/+* and *Lep-/-* mice following cold exposure. Cold exposure also increased the expression of antidiuretic hormone arginine vasopressin (*Avp*) gene in both mutant and wild-type animals by 1.8 and 1.4 fold, respectively. Each of the genes expressed in parallel in *Lep+/+* and *Lep-/-* mice were validated by qRT-PCR (Fig 5B).

### Experiment 3: Regulation of *Npvf* expression in mouse hypothalamus under variable thermogenic conditions

**Fig 6:**
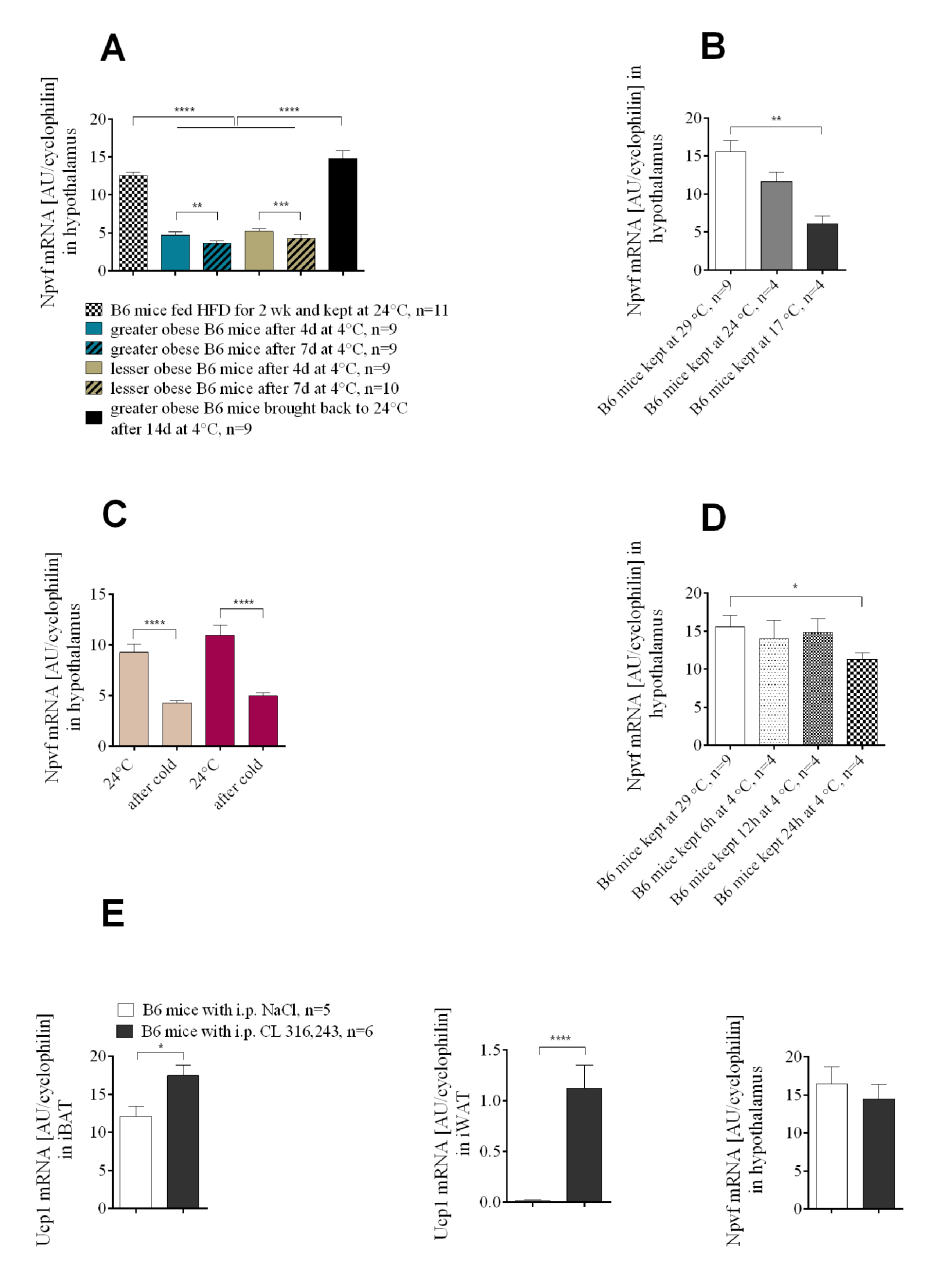
The expression of *Npvf* in the hypothalamus responds to reduced ambient temperature and time of exposure to the cold, but is not associated with the level of non-shivering thermogenesis in iBAT. Cold-induced changes in *Npvf* mRNA expression in greater obese and lesser obese B6 mice (A). Ambient temperature-dependent expression of *Npvf* mRNA in hypothalamus of chow-fed wild-type B6 mice (B). Cold-induced changes in *Npvf* mRNA expression in mutant *Ucp1-/-* and normal control *Ucp1+/*? mice (C). Time-course of changes in the level of *Npvf* mRNA in hypothalamus under low temperature conditions (D). Changes in the expression of *Ucp1* mRNA in brown and white adipose tissue and *Npvf* mRNA in hypothalamus (E) in response to 7 days of either CL 316,243 or saline administration at 29°C in B6 mice. Data are expressed as mean ± SEM. *, significant differences between mice (*t* test, *, *P* < 0.05; **, *P* < 0.01; ****, *P* < 0.001).

To further investigate a potential role for Npvf in food intake as a function of cold, we determined its expression in the hypothalamus of mice with different levels of dietary-induced obesity following cold exposure (Figs 1A and S1A). Similar to the experiment with *Lep+/+* and *Lep-/-* mice, *Npvf* expression was suppressed in both greater and lesser obese mice after the temperature shift from 24 to 4°C, but its expression was not associated with either adiposity or food intake (Fig 6A). Increasing the duration of cold exposure at 4°C from 1 to 7 days gradually amplifies the reduction in *Npvf* mRNA levels. In an independent experiment DIO mice that were maintained at 4°C for 14 days and then returned to 24°C for 25 days restored their levels of *Npvf* mRNA to that initially observed at 24°C (Fig 6A). *Npvf* mRNA expression in hypothalamic tissue showed a positive correlation with ambient temperature. Mice kept for 14 days at thermoneutrality (29°C) had higher expression of *Npvf* mRNA in hypothalamus than mice maintained at 24°C. Similarly, 2 weeks at 17°C resulted in a reduction of mRNA expression to levels below that observed at 24°C (Fig 6B).

Although modulation of *Npvf* precursor mRNA occurs during cold-stimulated thermogenesis, an involvement of Npvf in the regulation of non-shivering thermogenesis in brown fat is unlikely. Down-regulation of *Npvf* mRNA was not influenced by the absence of UCP1 protein (Fig 6C). Acute exposure to the cold requires an immediate response for heat generation and leads to immediate UCP1 production in BAT and WAT. A separate experiment performed to illustrate time-course of changes in the expression of *Npvf* under low temperature conditions showed that significant suppression in the amount of *Npvf* mRNA does not occur before 12h at 4°C; a significant decrease in the accumulation of *Npvf* mRNA in hypothalamus is found after 24h at 4°C compared to 29°C (Fig 6D). Moreover, one week administration of β3-adrenergic agonist CL 316,243 (1mg/kg of body weight) did not result in the suppression of *Npvf* mRNA in hypothalamus compared to saline-treated control mice (Fig 6E), providing evidence that changes in expression of the *Npvf* gene are not linked to heat production or brown adipocyte induction in peripheral β3-AR-expressing tissue targets.

### Expression of CNS and peripheral genes associated with energy metabolism

Genes associated with food intake in the hypothalamus, thermogenesis in the adipose tissue, and lipid metabolism in the liver and adipose tissues were analyzed by qRT-PCR. No patterns in gene expression could illuminate mechanisms associated with the phenotypes described above (see Supplement; S3A and S3B Fig for thermogenic genes in iBAT and iWAT, S4A and S4B Fig for neuropepetides of feeding behavior, and S5A Fig for genes of fatty acid metabolism in the liver, S5B Fig in iBAT and S5C Fig in iWAT).

## Discussion

We show that the total energy expended by a mouse from food intake and endogenous energy reserves to sustain thermogenesis during cold exposure is independent of the degree of obesity in the animals. This is true in genetically obese *Lep-/-* mice, chow-fed wild-type mice (Fig 3F) and in wild-type mice and B6.*Ucp1-/-* with variable levels of DIO (Figs 1G and 4E, respectively). However, in chow-fed mice the energy that is necessary to sustain a thermogenic program to maintain body temperature in the cold comes exclusively from feeding, as observed by others [20,26]; whereas in a wild-type mouse with diet-induced obesity induced by a high-fat diet, the fuel to support thermogenesis is obtained from endogenous energy reserves (mostly fat) and food intake. In DIO mice the source of energy required to maintain body temperature during cold exposure is determined by the degree of obesity. In DIO mice the energy reserves in fat mass are not privileged or restricted as those in a normal wild-type mouse maintained on a low-fat chow diet, rather they are utilized in proportion to their absolute levels. DIO mice with the highest levels of stored fat immediately mobilize fat, subsequently as these reserves become depleted, food intake becomes progressively a larger contributor to the fuel mix. In contrast, those mice that are at the other end of the DIO spectrum, the lesser obese mice, will preferentially increase food intake and use less of their endogenous fuel reserves to support thermogenesis. An important finding is that wild type mice with high levels of adiposity behave in response to cold exposure by the utilization of available energy sources in a manner that is independent of hormonal status, i.e. leptin and insulin. Serum leptin levels measured before cold exposure indicated that leptin resistance should have been higher in the greater obese mice than in the lesser obese, predicting a defense of adipose stores and higher food intake in greater obese mice. However, from the very beginning of cold exposure a defense of the fat status in mice with leptin levels predictive of leptin resistance was not observed. In fact the opposite was observed, with food intake reduced and fat utilization increased in the greater obese mice compared to the lesser obese.

Interestingly, our observations on body composition-dependent differential fuel selection occurring during cold exposure in DIO mice parallels findings in exercising human subjects (32). Moderate to intense physical activity performed regularly and on a long-term basis by lean individuals is compensated for by a corresponding change in food intake while body mass is maintained. On the other hand, obese individuals with excess fat storage do not significantly increase food intake and loss of body fat occurs as a consequence [27]. A return of mice fed a HFD from 6 to 24°C leads to a decrease in food intake and increase in adiposity characteristic of their phenotype on a high fat diet (S2A and S2B Fig). We previously observed the same response of mice fed a high fat diet when energy balance was interrupted with food restriction [18]. Accordingly, mice do not assume increased levels of food intake transiently acquired when they are in the cold, rather food intake is set by the requirements for heat production as originally hypothesized by Brobeck [28]. The long-term defense of body weight in humans and mice has been described and discussed as a consequence of under- and over-feeding [29]; however, mechanisms associated with a negative energy balance resulting from reduced energy intake during dieting may be different from increased energy expenditure in response to cold-induced energy expenditure, since the latter condition is supported by increased food intake and the neutralization of insulin and leptin resistance [2,30].

Wild-type B6 (*Lep+/- or Lep+/+*) mice fed a low fat chow diet exhibited almost no change in endogenous energy reserves, that is, lean mass or fat mass when the ambient temperature was reduced from 24 to 6°C, but they increased food intake. This observation fits with the thermostatic theory proposed by John Brobeck in the late 1940s, which relates the regulation of body temperature to the control of feeding behavior [28]. Brobeck summed up his theory by saying: “…animals eat to keep warm and stop eating to prevent hyperthermia”. In the present study, the wild-type B6 mouse maintained energy balance and body composition on a normal diet, when exposed to the cold, by increasing calorie intake. Importantly, the *Lep-/-* mouse behaved in the same manner, it adapted to the cold by increasing food intake in a manner quantitatively indistinguishable from the normal B6 mouse and it preserved its endogenous energy reserves. The β-oxidation of fat stores of *Lep-/-* mice is not an option for fuel to maintain body temperature [31] and this is a major factor in cold intolerance of leptin-deficient mice during acute exposure [32]. *Lep-/-* mice sensed that existing fat stores were unavailable and compensated by increasing food intake in a leptin-independent manner. This feeding behavior in the cold underscores the inability of mice with leptin-deficiency to utilize endogenous fat reserves; furthermore it also shows that in the face of a cold challenge fuel for thermogenesis must come from food intake. On the other hand, the wild-type mouse on a low fat chow diet can access its energy reserves in an acute situation, but quickly turns to increased food intake to maintain energy balance. This similarity in the metabolic response to the cold environment between normal and leptin-deficient mouse suggests that leptin is not important for the acute thermogenic phenotype in the *Lep-/-* mouse, nor for the regulation of food intake during cold exposure by normal wild-type mice fed a chow diet.

Mice with mutations to leptin and the leptin receptor have a thermogenic phenotype in which body temperature drops about 10°C in about 4 hours at an ambient temperature of 4°C [21]; however, as illustrated in Fig 3D they can adapt to the cold when it is gradually reduced. A key feature of cold-induced thermogenesis in normal animals is the increase in food intake that occurs over and above the increase in food intake necessary to support nutrition [33–36]; as exemplified by the remarkable boost in food intake that occurs in lactating females exposed to the cold [26]. This suggests that central mechanisms controlling food intake, as related to nutrition, growth and body composition, may be independent of those associated with cold-induced thermogenesis. A similar idea has been put forth by Speakman and Krol [37], but with a necessary role for leptin in the cold-induced food intake, which we did not see, nor was a role for leptin proposed by Melnyk and Himms-Hagen [6]. We had observed previously, as did Coleman [20], that *Lep-/-* mice exposed to the cold further increased food intake above that normally occurring in these mice [19]. This preliminary observation has been extended in this study to show that this hyperphagia, which is above that normally occurring in *Lep-/-* mice fed a chow diet, is very similar in magnitude and kinetics to that occurring in *Lep+/+* mice. Accordingly, mechanisms controlling cold-associated food intake in *Lep-/-* mice are independent of leptin-based regulation of food intake. We tested further the role of leptin in regulating thermogenesis during cold exposure in wild-type DIO mice. Plasma leptin and insulin levels in DIO mice of this study are remarkably similar to mice described in previous studies that were leptin resistant [38]. If the mobilization of fuels for cold-induced thermogenesis in DIO mice is controlled by the leptin resistance at the time of cold exposure, then one would predict that food intake would be high and mobilization of endogenous fat stores would be low. However, within one day of exposure to the cold the opposite phenotype was observed in DIO mice: food intake was low and fat mobilization was high. Even 4 days after cold exposure plasma levels of leptin were not significantly different from those at 24°C; only after 7 days in the cold were the levels of leptin significantly reduced (Fig 2D). Additional leptin administered intraperitoneally to DIO and lean mice did not affect the observed pattern of energy substrate utilization in the cold (Fig 2E). This data additionally suggests that the mechanism controlling food intake during acute cold exposure is independent of leptin signaling. Chronic cold adaptation may involve leptin by another mechanism [19].

The primary motive driving this study was to explore the feasibility of using cold exposure as an anti-obesity strategy. Human studies on brown fat show dramatic inter-individual differences in brown adipocyte content and BAT activity [39,40]. Thus, it is important to assess how the cold-stimulated effect of body weight reduction is influenced when the capacity for thermogenesis in brown fat is variable. The extent to which BAT-mediated adaptive thermogenesis could account for variability in substrate utilization in the reduced ambient temperature is also not known. For these reasons we evaluated the phenotype of DIO *Ucp1-/-* mice lacking functional brown fat. *Ucp1-/-* mice are sensitive to the cold; however, they can adapt to the cold if the ambient temperature is gradually reduced [24,41].Therefore, if UCP1 is essential to the thermogenic process, then in its absence the capacity for heat production from brown fat would be severely suppressed and we could expect effects on food intake and or the utilization of endogenous fuels that would differ from the wild-type mouse. As expected, when the ambient temperature was reduced average food intake was higher in the *Ucp1-/-* mice than in control mice, because these mice are less obese when fed a high-fat diet and they burned less of their endogenous reserves compared to normal *Ucp1+/*? mice with the greater obese phenotype (Fig 4B and 4D). Thus, there does not seem to be any difference in the pattern of utilization of endogenous food reserves or food intake between UCP1-deficient and wild type mice, provided that these mice have similar adiposity phenotypes as occurs with the lesser and greater obese mice.

QRT-PCR analysis of the expression of several genes in the hypothalamus encoding neuropeptides implicated with food intake did not provide evidence for the involvement of any of the neuropeptides associated with food intake with the possible exception of CART and POMC which have expression reduced by 30% and 50% in *Lep+/+* only. However, a microarray analysis of gene expression in *Lep-/-* and *Lep+/+* mice at 24 and 6°C showed that the expression of neuropeptide VF precursor was decreased 4-fold during cold exposure, and a similar level of down-regulation for this gene was observed for all three of the genetic models we have studied. In rodent brain, the sequence of the *Npvf* precursor gene predicts two–RFamide peptides: RFRP-1 and RFRP-3, also named NPSF and NPVF [42,43]. There is an expanding body of evidence for a role of various–RFamide peptides in the modulation of nociception, hormone secretion, reproduction or blood pressure [44–46]. Finally, although little is known of the functional significance of this particular biological effect, various–RFamide peptides were able to illicit a transient 10–300% induction or suppression of food intake in chicks, rats or mice after i.c.v. injection [44,47–50]. Moreover, food restriction or deprivation, both stimulating hunger and food hoarding, have been shown to be positively correlated with activation of RFRP-3 cells in the DMH of Syrian hamsters [51]. Effects on thermogenesis are unknown. From a functional viewpoint, specific expression of *Npvf* mRNA in the rodent central nervous system is restricted to a population of neurons localized between dorsomedial hypothalamic (DMH) and ventromedial hypothalamic (VMH) nucleus [42,43,52,53], which is consistent with a putative role in feeding or thermogenic processes [54]. Our observations on lack of association between leptin status and regulation of *Npvf* mRNA in the cold demonstrate that Npvf system in hypothalamus is unlikely to be leptin responsive, which is in agreement with a recent study, where no evidence for leptin signaling after leptin injection or detection of leptin receptors in RFRP3 expressing neurons was found in mice hypothalamus [55]. Thus, the reduction in *Npvf* expression at lower temperature when a higher level of energy expenditure (EE) is required suggests that reduction of *Npvf* releases a brake on EE. Although increased food intake provides the fuel for the increase in EE in lesser obese mice, endogenous fat provides the fuel in greater obese mice. Since *Npvf* is similarly suppressed in both lesser and greater obese mice, neither endogenous substrate or food intake per se are the signals associated with *Npvf* expression levels. Lack of association between body energy reserves and hypothalamic *Npvf* expression was also shown in the recent study in which no significant difference in *Npvf* mRNA was detected between mice fed high-fat and low-fat diet for 20 weeks [55].

A motive for conducting our experiment was to establish in a mouse model the effects of cold on substrate utilization and long-term effects of cold exposure on food intake after a return to ambient temperature. This study clearly showed that upon cold exposure obese mice fuel their increase in energy expenditure with endogenous fat supplies, whereas lean mice increase food intake. An analysis of three mouse models of obesity suggests that reduced ambient temperature is effective in reducing diet-induced obesity without long-term compensatory increases in food intake. Whether humans will behave in a similar manner needs to be determined. The second part of our study uncovered evidence for a new hypothalamic signaling pathway, involving the *Npvf* gene, that is regulated in cold-activated thermogenesis. We will work towards determining whether a similar signaling pathway is present in humans.

## Materials and Methods

### Animals

Breeding pairs of C57BL/6J.*+/+*, C57BL/6J.*Ucp1+/-* and C57BL/6J.*Lep+/-* mice were obtained through the generosity of Dr. Martin Klingenspor of the Technical University of Munich, Germany. All procedures concerned with breeding, housing, maintenance and experimental treatment of the mice were approved by the Local Animal Care and Use Committee for University of Warmia and Mazury, Olsztyn. Guidelines for animal experiments followed EU Directive 2010/63/EU.

### Experiment 1: Energy expenditure during cold exposure of mice with DIO

The goal of this protocol was to generate a series of mice with a range of adiposities by use of a high-fat diet to determine the effects of cold exposure on changes in food intake and endogenous energy stores. Breeding pairs of C57BL/6J+/+ mice were housed at standard temperature (24±1°C) and maintained in ventilated rooms under a standard-day photoperiod (12:12-h light-dark period, lights on from 0700 to 1900 h) with free access to low-fat diet (PicoLab Rodent Diet 20, LabDiet 5053, 11.9 kcal % fat) and water. At 21 days of age male progeny were weaned and housed in groups of 3–5 in plastic cages with fresh sawdust bedding. Body weight and body composition by NMR (Bruker, BioSpin, Germany) were monitored until mice were 8 weeks of age, at which time mice were individually housed and divided into two nutritional groups matched for similar mean body mass and body fat content to form the lesser and greater obese groups. By 8 weeks of age, when mice were still on a low-fat diet, their body weights ranged from 19.2–25.5g (Fig 1A). Based on the NMR analysis, the distribution in body weights was mainly caused by differences in fat mass, with only a small contribution from fat free mass The greater obese group was fed a high-fat diet (AIN-76A with 33% hydrogenated coconut oil, 58 kcal % fat) from the 8^th^ to 16^th^ week to establish a range of mice with a higher adiposity index. The lesser obese would continue to be fed the low-fat diet (PicoLab Rodent Diet 20, LabDiet 5053, 11.9 kcal % fat) for 7 weeks and then the high fat diet for the 16^th^ week. Mice with body weights and fat mass ranging from 24.4 to 43.8g and 3.7 to 18.8g, respectively, were formed (Fig 1A–1D). The aim of such a dietary intervention was to establish as broad a range in adiposity as possible between two groups of mice and at the same time to induce metabolic adaptations associated with high-fat feeding in the lesser obese mice at the time of cold exposure. In addition to having mice with a variation in adiposity, food intake of the lesser and greater obese mice was measured for the 16^th^ week, just prior to being exposed to the cold. Food intake, initially varied in the lesser obese mice when presented with a highly palatable high-fat diet for the first time; however, on the last day before cold exposure, there was no significant difference in food intake between the lesser and greater obese mice (S1E Fig).

In order to determine the relative contribution of food intake and endogenous energy reserves to fuel cold-induced thermogenesis, individually housed mice in the lesser and greater obese groups of mice were transferred to a cold room at 4°C for either 4 or 7 days. Food intake (high-fat; AIN-76A) and body weight were measured daily and body composition was analyzed by NMR at the end of the cold exposure. To calculate daily energy expenditure in the cold coming from endogenous and exogenous energy sources, fat mass and fat free mass measured after cold exposure were subtracted from fat mass and fat free mass measured before cold exposure and divided by number of days spent in the cold; average daily food intake measured at 24°C was subtracted from average daily food intake measured at 4°C. Energy values in kJ for g of fat mass or fat free mass were calculated as follows: 4.18 kJ/kcal × (9 or 4 kcal/g, respectively). Energy values in kJ for g of low-fat chow diet or high-fat diet were calculated as follows: 4.18 kJ/kcal × (3.07 or 5.44 kcal/g, respectively).

To observe the effects of cold-induced hyperphagia on DIO mice that were returned to an ambient temperature of 24°C, adult C57BL6/J+/+ mice were fed a high-fat diet (AIN-76A) from 8 to 16 weeks of age then transferred to 4°C until food intake stabilized over a course of 16 days. Mice were returned to an ambient temperature of 24°C and the suppression of food intake was monitored for an additional month.

To assess the effects of leptin treatment (1μg/g BW twice a day) on utilization of energy fuel coming from endogenous reserves or food intake, we measured daily changes in food intake, body weight and composition in 8 week-old lean B6 male mice fed chow diet and 16 week-old DIO B6 male mice fed HFD. Leptin was administered for 4 days at 24°C and for an additional 4 days at 4°C.

### Experiment 2: This experiment had parts (a) and (b)

#### Part (a): The effects of leptin deficiency on cold-induced energy expenditure

Eight week-old wild-type C57BL/6J.*Lep+/+*, (n = 13), heterozygous C57BL/6J.*Lep+/-*, (n = 10) and homozygous null C57BL/6J.*Lep-/-*, (n = 19) mice of both sexes were individually housed in an environmentally controlled chamber (EHRET GmbH, Emmendingen, Germany) and fed standard rodent chow pellets (LabDiet 5053, 11.9 kcal % fat) during the whole experiment. Housing conditions (photoperiod, air changes) were the same as in *experiment 1*. After 3 days of habituation to the chamber environment (24°C), food intake at 24°C was recorded during 3 consecutive days using high-precision food weighting sensors (PhenoMaster System, TSE Systems GmbH, Bad Homburg, Germany), then, the temperature in the chamber was reduced by 3°C/day to 6°C at which time mice were kept at 6°C for additional 2 days. Since *Lep-/-* mice cannot tolerate an acute reduction in ambient temperature to 6°C, this gradual reduction in ambient temperature was implemented to adapt the mice to the cold [20,21]. Body composition was analyzed by NMR before and after the cold test.

#### Part (b): The effects of UCP1 deficiency on cold-induced energy expenditure

Both backcross (C57BL/6J.*Ucp1-/-* x C57BL/6J.*Ucp1+/-*) and intercross (C57BL/6J.*Ucp1+/-* x C57BL/6J.*Ucp1+/-*) matings were used to generate C57BL/6J.*Ucp1-/-* mice together with heterozygous and homozygous normal (wild-type) controls [23]. Male mice were fed chow diet (LabDiet 5053, 11.9 kcal % fat) until 8 weeks of age. Obesity was induced in mice by feeding them a high-fat diet (AIN-76A, 58 kcal % fat) from 8 to 16 weeks of age. At 16 weeks of age mice were transferred to the temperature-controlled chamber (EHRET GmbH). Housing conditions, the protocol for exposing the mice to the cold and phenotyping of adiposity and food intake was the same as that described for part (a).

Energy expenditure was calculated as described for *experiment 1*.

### Experiment 3: Regulation of *Npvf* expression in mouse hypothalamus under variable thermogenic conditions

All mice used in the following studies were adult wild-type C57BL/6J+/+ mice. Mice were placed in individual cages with free access to food (LabDiet 5053) and water. At the end of each experiment mice were sacrificed and hypothalamus was dissected in order to measure the level of *Npvf* mRNA expression. To establish the influence of different ambient temperature on *Npvf* expression mice were kept in climate-controlled rodent incubators set to 29 and 17°C for the period of weeks prior to sacrifice. Additional experiment was performed to observe the kinetics of changes in the level of *Npvf* mRNA in the cold. All mice used in this study were first allowed to acclimate to 29°C for 2 weeks before cold challenge. Temperature of the housing unit was then transitioned from 29 to 6°C and mice were cold-challenged for 6, 12 or 24h. To evaluate the effects of the β3 adrenergic receptor agonist on *Ucp1* and *Npvf* thermoneutrally acclimated mice were injected subcutaneously with 1 mg/kg BW/day CL 316,243 or saline for 7 days.

### Metabolic and molecular assays

Mice were anesthetized by the solution of ketamine, xylopan and chlorpromazine (26.6 mg/ml, 1.67 mg/ml and 0.53 mg/ml, respectively, 40μl/10g body weight) and the blood was collected through heart puncture to EDTA coated tubes. After decapitation, interscapular brown adipose tissue depot (iBAT), inguinal white adipose tissue depot (iWAT) and the liver were removed, rapidly frozen in liquid nitrogen and stored at -80°C for subsequent preparation of total RNA. To isolate the whole hypothalamus, the brain was removed and placed on an ice-cooled glass plate with the cortex facing down. The hypothalamus was dissected along the following boundaries: laterally 2 mm either side of the third ventricle, 2 mm dorsally from the base of the brain and rostrocaudally from the optic chiasm to the posterior border of the mammillary bodies. The dissected hypothalami were stored at -80°C until further analysis. The blood was centrifuged for 10 min at 3,000 g, 4°C. Plasma was removed and stored at -80°C until assayed.

### Plasma hormones and metabolites

Plasma insulin and leptin were measured by enzyme-linked immunosorbent assay with commercial kits (Wide range mouse insulin immunoassay kit, Biorbyt Ltd., Cambridge, UK; Mouse/rat leptin ELISA kit, Phoenix Pharmacuticals, Inc., Burlingame, CA, United States, respectively). Assessment of FFA in plasma was performed with plasma non-esterified free fatty acid detection kit (Zenbio, Inc., Research Triangle Park, NC, United States).

### Quantitative real-time PCR

Total RNA was isolated from adipose tissue, liver and hypothalamus using TRI Reagent and BCP phase separation reagent (Molecular Research Center Inc. Cincinnati, OH, United States). RNA was further purified by using the RNAeasy minikit (QIAGEN, Valencia, CA, United States) and stored at -80°C in RNase-free H_2_O with addition of SUPERase-In (Ambion, Austin, TX, United States) for RNase protection. Quality and quantity of RNA was determined using UV spectrophotometry (Nanodrop) and agarose gel visualization of intact RNA. Quantitative RT-PCR using TaqMan probes and primers (Applied Biosystems, Foster City, CA, United States) was performed with standard curves generated using pooled RNA isolated from corresponding tissues collected from eight 8 week old C57BL/6J.*+/+* mice. Probe and primer sequences used to perform the analyses are available upon request. All the gene expression data were normalized to the level of cyclophilin b.

### Microarray analysis of gene expression in the hypothalamus of *Lep-/-* and *Lep+/+* mice

Total RNA was isolated from the hypothalamus of 8 *Lep-/-* and 8 *Lep+/+* mice maintained at 24 and 6°C, as described above. RNA with RNA Integrity number higher than 8.5 (Agilent 2100 Bioanalyzer, Agilent Technologies, Santa Clara, CA) was used for microarray analysis of each individual mouse. RNA was amplified, labeled and hybridized onto chips containing over 56,000 probes of mouse genes (Agilent Single Color SurePrint G3 Mouse GE 8x60K Microarray Kit, G4852A, Agilent Technologies) according to manufacturer’s guidelines. Agilent Feature Extraction software was used for array image analysis. Absolute and comparative analyses were performed using the GeneSpring GX 10 (Agilent Technologies). Quality control filtering after quantile normalization resulted in approximately 33,000 probes. Probes that were not above microarray background signal or whose sequences could not be mapped to Ensembl transcripts were discarded. Fold change of gene expression was calculated based on the normalized signal values. Genes were considered significantly down-regulated or up-regulated if the fold-change was less than -1.4 or greater than 1.4, respectively, and the FDR-corrected *P*-value was less than 0.05. To validate the reliability of the results obtained from the microarray analysis, we performed qRT-PCR for all genes of interest.

### Statistical analysis

Graphs were created with the GraphPad Prism Software (Version 6.0, GraphPad Software, Inc.; La Jolla, USA). All data sets were analyzed using Student’s test for groups (GraphPad Prism Software). Data are presented as means ± SEM. Differences between the means for all tests were considered statistically significant if *P* < 0.05.

## Supporting Information

S1 Fig Changes in endogenous substrate utilization and food intake associated with cold-induced thermogenesis in mice with variable levels of DIO. Time-course of changes in body weight during cold exposure (A). Body weight, fat mass, and fat free mass before and after 7 days at 4°C (B). Daily changes in food intake during 7 consecutive days at 4°C (C). Correlations between adiposity index determined before cold exposure and the change in daily energy consumption during 4 days in the cold (D). Daily changes in food intake during the last week (week 16^th^) before cold exposure (E). Data are expressed as mean ± SEM. *, significant differences (*t* test, *, *P* < 0.05; **, *P* < 0.01; ***, *P* < 0.005; ****, *P* < 0.001).
(TIF)

S2 Fig Cold induced food intake returns to baseline when ambient temperature returns to 24°C. Changes in food intake (A) and body weight (B) in DIO B6 mice that were brought back to standard temperature after cold exposure.
(TIF)

S3 Fig Cold-activated thermogenesis in DIO mice. Changes in the expression of genes associated with thermogenic processes in iBAT (A) and iWAT (B) before and after 4 and 7 days at 4°C in B6 mice with variable levels of DIO. Data are expressed as mean ± SEM. *, significant differences (*t* test, *, *P* < 0.05; **, *P* < 0.01; ***, *P* < 0.005; ****, *P* < 0.001).
(TIF)

S4 Fig Changes in the expression of genes encoding for neuropeptides associated with feeding behavior in hypothalamus before and after 4 and 7 days at 4°C in DIO B6 mice (A) and in mutant *Lep-/-* and control wild-type *Lep+/+* mice before and after the cold acclimatization protocol (B). Data are expressed as mean ± SEM. *, significant differences (*t* test, *, *P* < 0.05; **, *P* < 0.01; ***, *P* < 0.005).
(TIF)

S5 Fig Cold-induced changes in the expression of genes associated with lipogenesis in the liver (A), iBAT (B) and iWAT (C) before and after 4 and 7 days at 4°C in DIO B6 mice. Data are expressed as mean ± SEM. *, significant differences (*t* test, *, *P* < 0.05; **, *P* < 0.01; ***, *P* < 0.005; ****, *P* < 0.001).
(TIF)
