# Correction: Npvf: Hypothalamic Biomarker of Ambient Temperature Independent of Nutritional Status

**DOI:** 10.1371/journal.pgen.1005431

**Published:** 2015-09-17

**Authors:** Julia Jaroslawska, Agnieszka Chabowska-Kita, Monika M. Kaczmarek, Leslie P. Kozak

In the Data Availability statement, the accession number is listed incorrectly. The correct accession number is: GSE 59517.
